# Comparative Evaluation of Liposomal Albendazole and Tablet-Albendazole Against Hepatic Cystic Echinococcosis: A Non-Randomized Clinical Trial

**DOI:** 10.1097/MD.0000000000002237

**Published:** 2016-01-29

**Authors:** Haitao Li, Tao Song, Yingmei Shao, Tuergan Aili, Ayifuhan Ahan, Hao Wen

**Affiliations:** From the State Key Lab Incubation Base of Xinjiang Major Diseases Research (2010DS890294) and Xinjiang Key Laboratory of Echinococcosis (HL, TS, YS, TA, AA, HW); Hepatobiliary & Hydatid Department, Digestive and Vascular Surgery Centre (HT, TA, AA, HW); and Department of Ultrasonography, First Affiliated Hospital, Xinjiang Medical University, Urumqi, China (TS, YS).

## Abstract

In this study, we aimed to compare the clinical efficacy of liposomal albendazole (L-ABZ) and tablet-albendazole (T-ABZ) for the treatment of human hepatic cystic echinococcosis (CE).

Sixty patients with single cyst (CE1) or daughter cyst (CE2) were included in this study and were nonrandomly divided into the L-ABZ group (n = 30, 10 mg/kg per day, p.o., b.i.d.) and T-ABZ group (n = 30, 12–20 mg/kg per day, p.o., b.i.d.), respectively. The treatment duration lasted for 6 months, during which dynamic follow-up was carried out to evaluate the clinical efficacy through calculating the total effective rates (TERs). Measurement data and numerous data were analyzed by the chi-square test. Two-sided tests were performed for all the statistical tests.

In our study, 2 patients were lost in the follow-up in the L-ABZ group. One patient was lost in the follow-up in the T-ABZ group, and 1 patient was withdrawal from the study due to receiving surgery. Significant difference was identified in the 3-month TERs of L-ABZ group and T-ABZ group (33.3% vs 76.7%, *P* < 0.05). Also, remarkable difference was noted in the 6-month TERs in the L-ABZ group and T-ABZ group (66.7% vs 93.3%, *P* = 0.01). No statistical difference was noticed in the incidence rate of adverse reactions in both groups (*P* > 0.05).

Based on our study, both T-ABZ and L-ABZ are effective for treating human CE. The TER in the L-ABZ group is superior to that of T-ABZ.

## INTRODUCTION

Cystic echinococcosis (CE) is a zoonosic disease caused by the infection by the larval stages of *Echinococcus granulosus*. To our best knowledge, the vast majority of infected population is located in the pastoral regions.^1^ Liver is the most commonly affected organ in patients infected by CE, and on some occasions, other organs are also affected such as lung, kidney, and spleen.^2^ Currently, benzimidazoles (BMZ), namely flubendazole, mebendazole, and albendazole representing the main category of drugs, have been considered as effective treatment options for CE. Among these drugs, tablet albendazole (T-ABZ) has been predominantly used in clinical practices.^3^ Despite it has been well acknowledged in treating CE, its clinical efficacy is still not validated by randomly controlled trails. Also, the efficacy of ABZ on the management of CE is controversial.^4^

To date, the efficacy of T-ABZ has been approved in treating CE as revealed by a large amount of clinical cases.^5^ However, the absorption of T-ABZ in intestinal tract was comparatively poor. Also, the drug concentration was lower in liver and lung, which undermined the treatment efficiency to some extent.^6^ Currently, no consensus has been achieved on the efficiency of systematic management of EC using T-ABZ as different drug dosage and management duration are used in different countries. Additionally, increased side effects seem to be inevitable under therapeutical doses.^7^

Recently, extensive studies have been carried out for the screening of drugs with high EC-killing efficacy and low toxicity. Nowadays, one of the major methods used to improve the medication efficacy is to increase the drug concentration in target organs. To be exact, prolonged bioavailability and enhanced clinical efficacy are recommended by changing the pharmaceutical dosage forms.^8^ In our previous study, liposomal albendazole (L-ABZ), a liposome-entrapped albendazole with high liposolubility and stability, has been developed to increase the biological availability and efficacy of ABZ *in vivo*.^6^ Its clinical efficacy has been approved by our subsequent studies at the terms of patient safety and treatment efficiency ever since its clinical application in 1998.^9–11^ However, there are some limitations in these studies such as a lack of control study. In this prospective study, we compared the clinical efficiency and safety data in CE patients treating by T-ABZ or L-ABZ, respectively.

## METHODS

### Ethics Statement

All the patients signed the informed consents; we also obtained informed consent from guardians on behalf of the minors/children enrolled in your study, consent on behalf of the children enrolled was written. The protocols were approved by the Ethic Committee of First Affiliated Hospital of Xinjiang Medical University with an approval no. of 20080731, which has been submitted to the Chinese Clinical Trial Registry. The complete date range for patient recruitment and follow-up were also approved by the Ethic Committee. The clinical trial registration no. is ChiCTR-TNRC-11001329.

### Patients

Patients admitted to our department from October 2009 to September 2011 were enrolled in this study. The inclusion criteria were as follows: (1) those aged from 14 years to 70 years; (2) patients diagnosed with CE according to the guidelines proposed by WHO-Informal Working Group on Echinococcosis,^12^ including CE1 (simply cyst), CE2 (multiple daughter cyst) and CE3 (cyst with detachment of laminated membrane); (3) those received pharmacotherapy for >3 months; (4) patients who were diagnosed using the rapid diagnostic reagent kits as the auxiliary diagnosis standard. The exclusion criteria included: (1) those with CE4 (heterogeneous or hyperechoic degenerative contents) and CE5 (calcified cysts); (2) those with severe single or complicated organ dysfunction; (3) those with severe clinical symptoms such as icterus, abdominal pain, and could not be managed by using with anti-parasitic drugs along; (4) course of treatment for patients who received antiparasitic medication of <3 months; or (5) patients with administration of 2 or more antiparasitic drugs simultaneously.

### Treatment

Sixty patients were nonrandomly divided into the L-ABZ group (n = 30, 10 mg/kg per day, p.o., b.i.d., drug approve no. 991215) and T-ABZ group (n = 30, 12–20 mg/kg per day, p.o., b.i.d., drug approve no. H12020496) with their own free will under the instruction of the doctors. The administration duration for each group was 6 months. All the drugs were taken after meal.

### Evaluation of the Treatment Efficiency

Three categories were set to evaluate the treatment efficiency including: (i) noneffective, those with no improvement or even deterioration (with an increase of ≥2 cm in diameter in the affected organ) of the clinical symptoms and vital signs; (ii) effective, those with gradual or significant alleviation in the symptoms and vital signs, or with no significant increase in the cysts or mass, or necrosis in the content of the cyst, or a decrease of daughter cyst of ≥ 50%, or a decrease of ≥ 2 cm in the diameter of the cyst and focus, or elimination of ≥ 2 focus; and (iii) cured, those with complete elimination of the symptoms, or a decrease of ≥ 50% in the diameter of the affected area and the cyst, or elimination of the focus, or calcification in ≥ 50% of the focus. For patients with 2 or 3 cysts, it is considered effective if >1 cyst is sensitive to the drug. Cured is defined as all the cysts are sensitive to the drug.

### Follow-Up

Dynamic follow-up was carried out in this study. Every 3 months, the subjects were required to return to our hospital, and received a series of laboratory and/or clinical tests such as routine blood test, hepatic and renal function determination, routine urine analysis, as well as abdominal ultrasound. The ultrasonography results were blindly reviewed by the physicians in the Department of Ultrasound. The follow-up lasted for 6 months.

### Statistical Analysis

Blind review was performed by the staff in the Epidemiology and Health Statistics Institution, Xinjiang Medical University. SPSS17.0 software and PEMS 3.1 software were used for the data analysis. Measurement data and qualitative data were analyzed by *t* test and chi-square test. The Wilcoxon rank sum test was carried out for the analysis of ranked data. Two-sided tests were performed for all the statistical tests. Univariate analysis was performed using rank test. Logistic regression analysis was used for the multivariate analysis. *P* < 0.05 demonstrated statistical difference.

## RESULTS

### Patient Information

**FIGURE 1:**
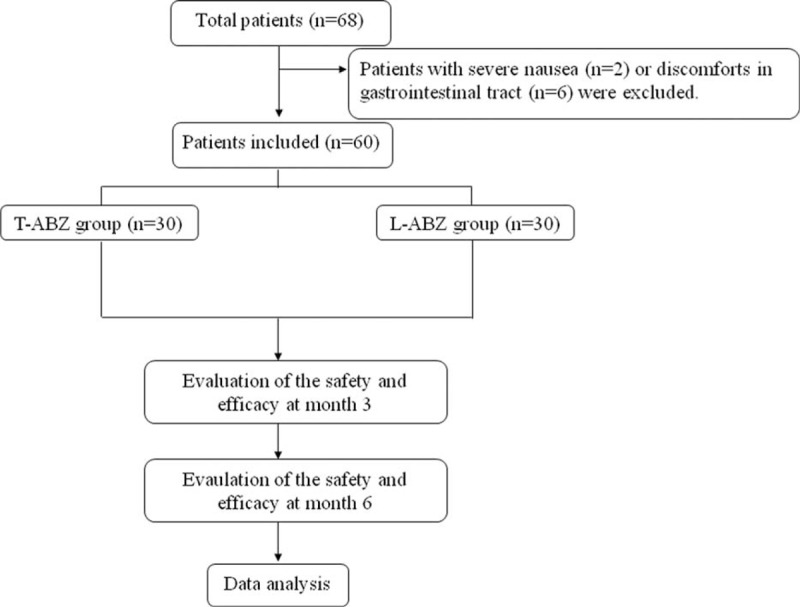
Flowchart. Number of subjects assessed for eligibility, enrolled, and nonrandomized to study medicine liposomal albendazole or tablet-albendazloe. Subjects were included in step-wise fashion with 30 subjects in each group.

A total of 60 patients were enrolled in this study. In the T-ABZ group, 30 patients (men: 14, women: 16, aged 14–70 years) were enrolled. The number of CE1 was 4, whereas that of CE2 was 26. For the L-ABZ group, 30 patients (men: 16, women: 14, aged 14–60 years) were enrolled. The number of CE1 was 6, whereas that of CE2 was 24 (Figure 1). No significant difference was noticed in the baseline information of the patients in both groups (*P* > 0.05, Table 1).

**TABLE 1:**
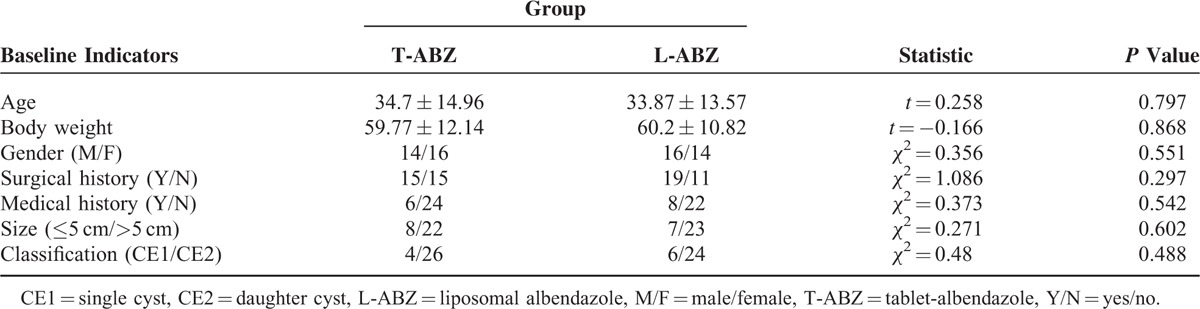
Comparison of Baseline Levels of T-ABZ Group and L-ABZ Group

### Total Effective Rate (TER)

Two patients were lost during the follow-up in each group, and the compliance in each group was 93.3% (28/30). For the patients lost in the follow-up, the clinical data obtained from the third month during the follow-up was used for the evaluation of the treatment efficiency. The TER was defined as the sum of effectiveness rate and cured rate.

**FIGURE 2:**
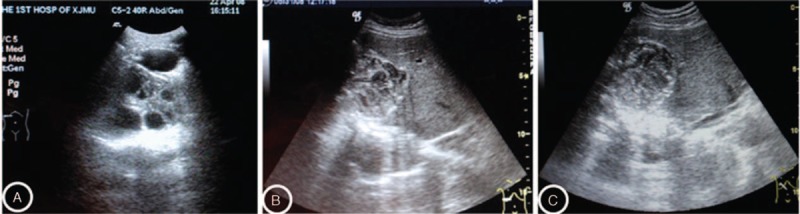
Ultrasound images of the CE lesions obtained from the baseline period (1A), 3 months (1B), and 6 months (1C) after administration of L-ABZ, respectively. The results indicated that complete response was noted at 6 months after administration of L-ABZ. CE = cystic echinococcosis, L-ABZ = liposomal albendazole.

**FIGURE 3:**
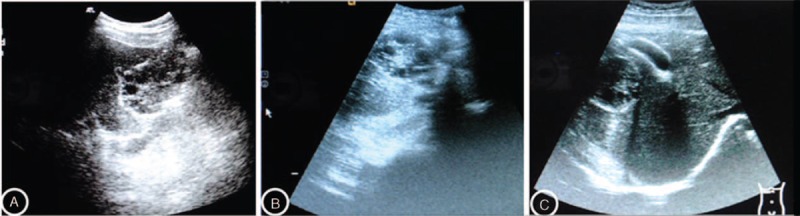
Ultrasound images of the CE lesions obtained from the baseline period (2A), 3 months (2B), and 6 months (2C) after administration of T-ABZ, respectively. The results indicated that after administration of T-ABZ was effective for treating CE. CE = cystic echinococcosis, T-ABZ = tablet-albendazole.

Both T-ABZ and L-ABZ were effective for treating CE as revealed by Figures 2 and 3. As shown in Table 2, significant difference was noted in the TER in the patients with administration of L-ABZ for 3 months compared with those with administration of T-ABZ for 3 months (76.7% vs 33.3%, *P* = 0.001). Compared with the patients of T-ABZ group, significant difference was noted in the TERs in patients of L-ABZ group with drug administration for 6 months (93.3% vs 66.7%, *P* < 0.05, Table 2).

**TABLE 2:**
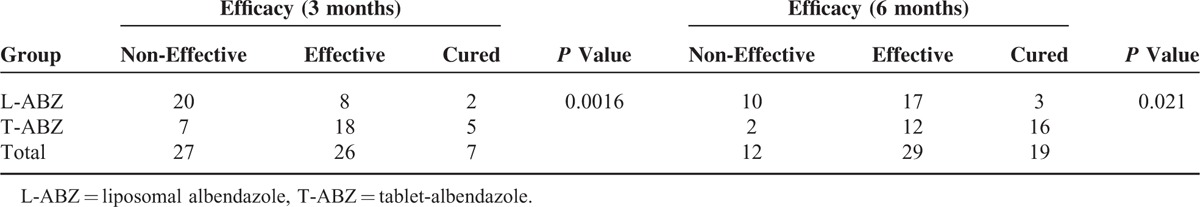
Comparison of the Clinical Efficacy at 3 Months and 6 Months After Chemotherapy

### Effects of the Cyst Diameter and the Type on the Treatment Efficiency

Logistic regression analysis was performed to analyze the correlation between the treatment efficiency and potential factors, including grouping (eg T-ABZ group or L-ABZ group), gender, age, body weight, history of surgery, medication, size of lesion (eg <5 cm or >5 cm), and type of lesion (eg CE1 or CE2). The results indicated that statistical difference was noticed in the 3-month TER or 6-month TER in the T-ABZ group compared with that of L-ABZ group. In addition, for the patients with various lesion sizes, the efficiency in the L-ABZ group was superior to that of T-ABZ group (Tables 3 and 4).

**TABLE 3:**
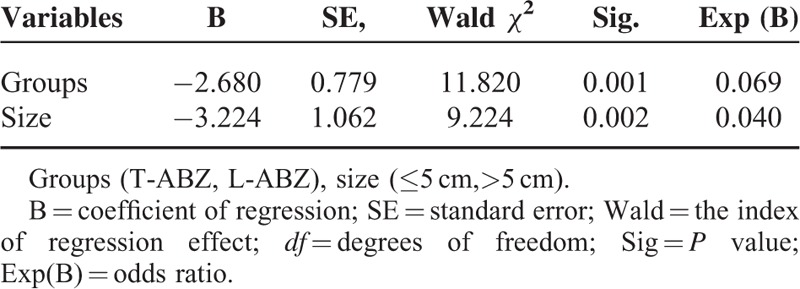
Significant Factors on the Clinical Efficacy 3 Months After Chemotherapy

**TABLE 4:**
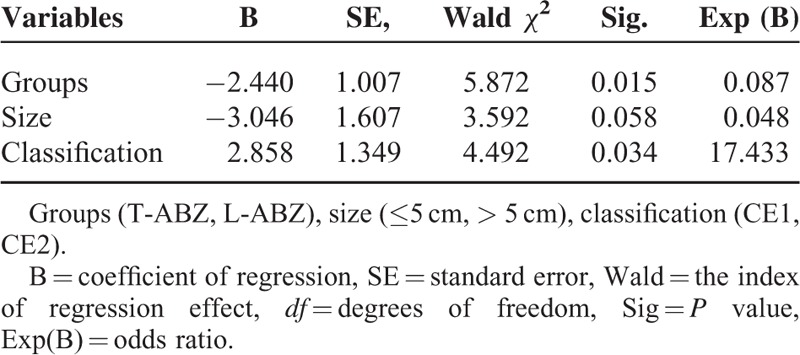
Significant Factors on the Clinical Efficacy 6 Months After Chemotherapy

### Side Effects

The side effects in the T-ABZ group included dizzy and gastrointestinal tract reactions, whereas that of the L-ABZ group included dizzy, diarrhea, and abdominal discomforts. An incidence rate of 10% was noticed in the side effects of both groups. Besides, a comparison was performed for the biochemical analysis results in both groups at 3 months and 6 months after treatment. Statistical difference was noticed in the serum aspartate aminotransferase (*P* = 0.046) and WBC (*P* = 0.016) at 3 months and 6 months after administration of L-ABZ as revealed by the paired rank test (Table 5). No statistical difference was observed in the alanine transaminase, total bilirubin, blood urea nitrogen, creatinine, white blood cell, red blood cell, hemoglobin, an platelet count, respectively.

**TABLE 5:**
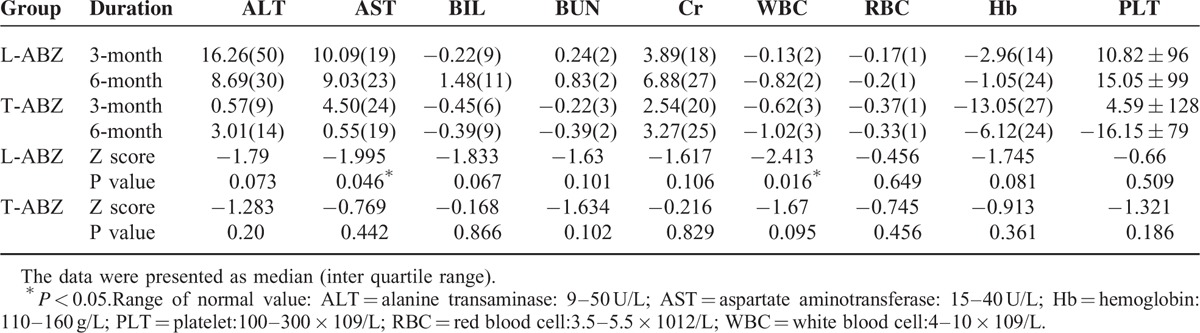
Biochemical Analysis Results in Patients of L-ABZ Group and T-ABZ Group at 3 Months and 6 Months After Treatment

## DISCUSSION

L-ABZ is an anti-parasitic drug developed in our hospital, in which ABZ is encapsulated in ultramicroscopic ball structure formed by lipid bilayer, and was gradually released in a controlled release manner. The drug showed low drug degradation *in vivo* and passive targeting capacity, which could improve the treatment efficiency and reduce the incidence of adverse reactions.^8^ As revealed by our results, 3-month TER and 6-month TER in the L-ABZ group were 76.7% and 33.3%, respectively. Those of T-ABZ group were 93.3% and 66.7%, respectively (*P* < 0.05). In addition, the dosage of L-ABZ administrated in the L-ABZ group was lower than that of T-ABZ group (10 mg/kg per day vs 12–20 mg/kg per day). Therefore, we concluded the clinical efficiency of L-ABZ was superior to that of T-ABZ in treating CE at 3 months with an OR of 0.069 (Table 3) and 6 months with an OR of 0.087 (Table 4), respectively. The reasons might be as follows: (1) L-ABZ with ABZ encapsulated by liposome could promote the absorption of drugs *in vivo*. Additionally, a large amount of phagocytes were observed in the liver and lung, which contributed to the passive tendency of liposome. (2) The gastrointestinal absorption of T-ABZ was comparatively lower *in vivo*, which resulted in poor outcome in the short term. In this study, randomized, double-blind trials could not be performed due to different drug dosages. Therefore, further random controlled trials (RCTs) should be carried out to validate its clinical efficiency according to the guidelines proposed by GCP/ICH.^13^

Generally, the treatment efficiency of patients with CE1 was superior to those with CE2.^5,14,15^ However, in our study, no statistical difference was identified in the treatment efficiency in patients with CE1 compared with those with CE2. We speculated that it may be related to the small sample size of CE1 patients, which may result in data bias. Previous studies indicated the size of cysts had an influence on pharmaceutical treatment efficiency in clinical practice.^5,16^ In our study, the treatment efficiency in patients with a cyst of <5 cm in diameter was comparatively higher than those with a cyst of ≥ 5 cm at the 3rd month and 6th month. Logistic regression analysis demonstrated the clinical efficiency of patients in L-ABZ was superior to these of T-ABZ, especially for the patients with larger cystic lesions. Moro P reported that patients with small (<7 cm in diameter) and isolated cysts responded best to the treatment, whereas patients with multiple compartments or daughter cysts were relatively refractory to treatment.^17^ Taken together, we speculated that patients with differing types and size of the cyst may response variously to the treatment.^18^

An incidence rate of 10% was noticed in the adverse reactions in the T-ABZ group and L-ABZ group, respectively. The adverse reactions in the T-ABZ group were dizziness, gastrointestinal tract reaction, as well as aberrant transaminase level as indicated by the hematology test. The adverse reaction in the L-ABZ group included dizziness, diarrhea, abdominal discomfort. Besides, statistical difference was noticed in the transaminase level and WBC count at 3 months (*P* = 0.046) and 6 months (*P* = 0.016) respectively between 2 groups. This indicated that drug accumulation may take place in the L-ABZ group. Such fact could increase the drug efficiency; however, it may also induce the possibility of adverse events. No severe adverse reactions were observed in our study. Previous reports showed long-term administration of albendazole, a broad-spectrum anti-parasitic agent, has been associated with cellular toxicity and side effects,^19,20^ including skin and mucous membrane diseases, nervous system diseases, digestive tract systematic disorders, cardiovascular diseases, urinary diseases, as well as hematological diseases such as anemia and leucocytopenia.^21,22^ The effective dose of T-ABZ was relatively higher as revealed in the previous study.^23^ However, the treatment efficiency and clinical safety were better in patients with administration of L-ABZ in this study. As we only evaluated the adverse reactions within 6 months, further clinical observations are needed to identify the potential adverse reactions in the long-term follow-up.

Currently, echinococcosis is still ignored in the world although it has caused great threats to the development of social economy and public health, especially in developing countries. Hundreds of years have been passed after the emergence of the diagnosis and treatment of echinococcosis, unfortunately, less attention has been paid accordingly.^24,25^ To date, no consensus has been achieved on the administration of benzimidazole, which has been considered as the effective medicine for treating cystic and alveolar echinococcosis.^7^ Furthermore, surgery or PAIR treatment rather than chemotherapy are preferred in treating echinococcosis.^26,27^ Extensive studies have been performed to identify the evidence regarding the role of albendazole for the treatment human echinococcosis, and preoperative administration of ABZ combined with surgery showed prior efficiency compared with surgery alone.^2,4^ Therefore, preoperative administration of L-ABZ is also suitable to reduce the incidence of relapse. Meanwhile, L-ABZ provided a new way for combined modality therapy in echinococcosis.^28^
